# Large-Scale Comparative Analysis of Eugenol-Induced/Repressed Genes Expression in *Aspergillus flavus* Using RNA-seq

**DOI:** 10.3389/fmicb.2018.01116

**Published:** 2018-05-30

**Authors:** Cong Lv, Ping Wang, Longxue Ma, Mumin Zheng, Yang Liu, Fuguo Xing

**Affiliations:** Institute of Food Science and Technology, Chinese Academy of Agricultural Sciences/Key Laboratory of Agro-products Quality and Safety Control in Storage and Transport Process, Ministry of Agriculture, Beijing, China

**Keywords:** aflatoxin B_1_, *Aspergillus flavus*, oxidative stress, transcriptome, gene regulation, eugenol

## Abstract

Aflatoxin B_1_ (AFB_1_), which is mainly produced by *Aspergillus flavus* and *Aspergillus parasiticus*, is the most toxic and hepatocarcinogenic polyketide known. Chemical fungicides are currently utilized to reduce this fungal contaminant, but they are potentially harmful to human health and the environment. Therefore, natural anti-aflatoxigenic products are used as sustainable alternatives to control food and feed contamination. For example, eugenol, presents in many essential oils, has been identified as an aflatoxin inhibitor. However, its exact mechanism of inhibition is yet to be clarified. In this study, the anti-aflatoxigenic mechanism of eugenol in *A. flavus* was determined using a comparative transcriptomic approach. Twenty of twenty-nine genes in the aflatoxin biosynthetic pathway were down-regulated by eugenol. The most strongly down-regulated gene was *aflMa*, followed by *aflI, aflJ, aflCa, aflH, aflNa, aflE, aflG, aflM, aflD*, and *aflP*. However, the expression of the regulator gene *aflR* did not change significantly and the expression of *aflS* was slightly up-regulated. The down-regulation of the global regulator gene *veA* resulted in the up-regulation of *srrA*, and the down-regulation of *ap-1* and *mtfA*. The early developmental regulator *brlA* was profoundly up-regulated in *A. flavus* after eugenol treatment. These results suggested a model in which eugenol improves fungal development by up-regulating the expression of *brlA* by the suppression of *veA* expression and inhibits aflatoxin production through the suppression of *veA* expression. Exposure to eugenol also caused dysregulated transcript levels of the G protein-coupled receptors (GPCRs) and oxylipins genes. A Gene Ontology analysis indicated that the genes that were highly responsive to eugenol were mainly enriched in RNA-binding functions, suggesting that post-transcriptional modification plays a pivotal role in aflatoxin biosynthesis. KEGG analysis showed that ribosome biogenesis was the most dysregulated pathway, suggesting that eugenol dysregulates ribosome biogenesis, which then interrupts the biosynthesis of Nor-1, Ver-1, and OmtA, and prevents aflatoxisomes performing their normal function in aflatoxin production. In conclusion, our results indicated that eugenol inhibited AFB_1_ production by modulating the expression of structural genes in aflatoxin pathway, fungal antioxidant status, post-transcriptional modifications and biosynthesis of backbone enzymes in *A. flavus*.

## Introduction

*Aspergillus flavus* is a saprotrophic filamentous fungus that occurs widely in agricultural and medical products (Liang et al., 2015). It contaminates many important agro-products including peanut, maize, rice, cottonseed, sunflower seed, herbal material, and feeds (Cleveland et al., 2004; Liang et al., 2015). *A. flavus* produces many kinds of secondary metabolites including aflatoxin, cyclopiazonic acid, conidial pigment, aflatrem and kojic acid (Bennett and Klich, 2003; Hoffmeister and Keller, 2007). Of them, aflatoxin is the most toxic and hepatocarcinogenic compound (Squire, 1981). As a carcinogen, aflatoxin is estimated to cause up to 28% of the total global cases of hepatocellular carcinoma, the most common form of liver cancer (Wu, 2014; Xing et al., 2017). Moreover, aflatoxin leads to acute intoxication, immune-system disruption and growth impairment in children (Groopman et al., 2008).

Eugenol (4-allyl-2-methoxy phenol), a natural substance used as a food- flavoring agent, was first isolated in 1929 and its commercial production began in 1940 in the United States (da Silva et al., 2018). It is mainly extracted from *Syzygium aromaticum, Ocimum tenuiflorum, Pimenta racemosa, Zieria smithii*, and *Cassia fistula* although it can be produced synthetically (Jayashree and Subramanyam, 1999). As an allyl-phenol-type phenylpropanoid, eugenol is a pale yellow oil with clove odor and spicy taste (da Silva et al., 2018). Eugenol can be oxidized in a non-enzymatic manner, such as light via a one-electron pathway to a phenoxyl radical (ArO·), and subsequently to eugenol quinonemthide (QM) by light (Satoh et al., 1998; Choi et al., 2009). It is generally regarded as safe by the Food and Agricultural Organization (Opdyke, 1975), with an acceptable daily intake of up to 2.5 mg/kg body weight in humans (FAO, 1982) based on its non-mutagenic and non-carcinogenic properties [International Agency for Research on Cancer (IARC), 1985]. Eugenol is widely used in the pharmaceutical, food, agricultural and cosmetics industries because it exerts useful antimicrobial and antioxidant effects (da Silva et al., 2018). It also has other biological properties, including antiviral, anti-inflammatory, and anti-cancer effects, and inhibits platelet aggregation. Eugenol has previously been used as an AFB_1_ inhibitor.

Karapinar (1990) reported that the growth of *A. parasiticus* NRRL 2999 and *A. parasiticus* CBS 26027 was inhibited by eugenol at a concentration of 300 μg/mL (~1.83 mmol/L), and the production of aflatoxin particularly by *A. parasiticus* NRRL 2999 was enhanced by eugenol below 200 μg/mL (~1.22 mmol/L). Jayashree and Subramanyam (1999) found that aflatoxin production in *A. parasiticus* was inhibited by eugenol in a dose-dependent manner up to a concentration of 0.75 mmol/L without inhibiting fungal growth. They suggested that the anti-aflatoxigenic actions of eugenol were attributable to the inhibition of the ternary steps of aflatoxin biosynthesis, which involve lipid peroxidation and oxygenation. Nam and Kim (2013) demonstrated that eugenol inhibited aflatoxin biosynthesis through disrupting lipid peroxidation by reducing the microsomal activities of cytochorome P450, poly substrate monooxygenase (PSMO), and NADPH-dependent cytochorome C reductase. Using quantitative real-time PCR (q-PCR), Jahanshiri et al. (2015) indicated that eugenol strongly inhibited AFB_1_ production in *A. parssiticus* in the range of 15.07–98.0% in a dose-dependent manner. The expressions of major pathway genes such as *ver-1* (*aflM*)*, nor-1* (*aflD*), *pksA* (*aflC*), *omtA* (*aflP*), and *aflR* were significantly suppressed by eugenol at concentrations of 62.5 and 125 μg/mL (~0.76 mmol/L). In the meantime, Liang et al. (2015) also showed that eugenol (0.8 mM) inhibited AFB_1_ biosynthesis in *A. flavus* in Yeast Extract Sucrose (YES) broth by down-regulating the transcript levels of some key biosynthetic genes such as *aflP, aflM*, and *aflD*. Using a large-scale qPCR approach, Caceres et al. (2016) found that AFB_1_ inhibition by eugenol addition at 0.5 mM in a Malt Extract Agar (MEA) medium resulted in a complete inhibition of all but one gene of the AFB_1_ biosynthesis cluster. This phenomenon was modulated by the down-regulation of *aflR* and *aflS* expression and the over-expression of *veA* and *mtfA*, which are directly involved in regulating AFB_1_ cluster. However, the detailed molecular mechanism by which eugenol represses aflatoxin biosynthesis is still largely unknown.

RNA sequencing (RNA-Seq), a high-throughput sequencing technology with a low false-positive rate and high sensitivity, is widely considered a revolutionary tool for transcriptomics studies and has been used to investigate multiple eukaryotic transcriptomes (Wilhelm et al., 2008; Wang et al., 2009; Lin et al., 2013). Compared with the *de novo* assembly approach, a reference-based method is more accurate and sensitive. The *A. flavus* genome is a well-annotated genome available in NCBI with accession number AAIH00000000 (http://www.ncbi.nlm.nih.gov/nuccore/AAIH00000000). Transcriptome profiling of *A. flavus* has been used to investigate the effect of temperature and water activity (*a*_w_) on fungal development and aflatoxin biosynthesis (Yu et al., 2011; Zhang et al., 2014; Bai et al., 2015). Lin et al. (2013) profiled transcriptome of *A. flavus* to explore the inhibitory mechanism of 5-Azacytidine (5-AC) on fungal development and aflatoxin biosynthesis. They found that 5-AC affects fungal development through increasing the expression of *brlA* by depressing the expression of *veA* and affects aflatoxin production by suppressing *veA* expression (Lin et al., 2013).

To reduce aflatoxin contamination in foods, a number of strategies have been developed to either prevent fungal growth or block toxin production (Amaike and Keller, 2011). During planting, atoxigenic biocompetitive *A. flavus* and/or *A. parasiticus* strains or yeast are used to prevent fungal infection (Chang et al., 2012). During storage, chemicals, drying, natural products, and microorganisms have been applied to prevent fungal growth and aflatoxin production (Liang et al., 2015; Xing et al., 2017). Currently, chemical-based control remains the common measure used to control post-harvest aflatoxins contamination in a variety of foods. However, the application of chemicals not only increases the risk of toxic residues in foods but also often leads to fungal resistance (Isaac et al., 1999; Hua H. et al., 2014; Hua S. S. et al., 2014). Therefore, much effort has been directed in the recent years toward limiting the use of chemical fungicides in grains and foods. Essential oils from plants, such as phenolic and aldehydic compounds, acetate esters, and alcohols, provide an attractive alternative to inhibit fungal growth and aflatoxins formation because they efficiently eliminate aflatoxin, maintain food quality, are natural resources and are highly volatile (Wright et al., 2000; Bluma and Etcheverry, 2008; Roze et al., 2011).

In this study, the anti-aflatoxigenic mechanism of eugenol was determined with an RNA-Seq approach. A comprehensive view of the *A. flavus* transcriptome and the differentially expressed genes between eugenol treated and untreated samples were obtained. This study may extend our understanding of the inhibitory pathway of eugenol on aflatoxin biosynthesis and fungal development at the transcriptome level.

## Materials and methods

### Natural compound, fungal strain, and culture conditions

Natural eugenol (99% purity) extracted from clove buds was purchased from Xue-Song Company (Jiangxi, China) and dissolved in ethanol. The stock solution was stored at 4°C until use. The *A. flavus* strain YC-15 (Table S1) used in this study (Liang et al., 2015) was maintained in the dark on PDA medium (200 g boiled potato, 20 g dextrose, 20 g agar, 1 L) at 4°C. The conidia from a 7-day-old PDA culture were washed with 0.01% Tween-20 solution and counted with a hemocytometer. A suspension of 5 × 10^7^ conidia/mL was prepared. YES broth (20 g yeast extract, 150 g sucrose, 0.5 g MgSO_4_·7H_2_O, 1 L) was inoculated with the conidia at a final concentration of 10^6^ conidia/mL. The eugenol stock solution was diluted with ethanol to a concentration of 80 mM, and 500 μL of the diluted eugenol stock was added to 50 mL of YES broth, producing a final eugenol concentration of 0.80 mM. The control cultures were treated similarly but without eugenol. Each culture was incubated at 28°C in the dark for 5 days. The mycelia of *A. flavus* were then harvested. Each treatment was performed in triplicate.

### Preparation of cDNA and illumina sequencing

The cDNA preparation and Illumina sequencing were performed according to Zhang et al. (2014) with some modifications. *A. flavus* mycelia were harvested from YES broth for isolation of RNA. Total RNA was isolated using a Fungal RNA Kit (Omega, Norcross, USA) and genomic DNA was digested using RNA-free DNase I (Thermo Fisher Scientific, CA, USA). An Agilent 2100 Bioanalyzer and Nano Drop 2000 spectrophotometer were used to evaluate the integrity and concentration of RNA. The mRNA was isolated using oligo (dT)-attached magnetic beads. The isolated mRNA was mixed with fragmentation buffer and cleaved into small fragments (380 ± 50 bp) using divalent cations under elevated temperatures. The cDNA was synthesized using these cleaved RNA fragments as templates. After purification, these short cDNA fragments were subjected to an end-repair process with the addition of a single “A” base, and were then ligated to sequencing adaptors using the Illumina TruSeq DNA sample preparation kit. PCR amplification was performed using the qualified fragments as templates. Lastly, the libraries were sequenced using an Illumina HiSeq 4000 system.

### Clean reads and normalized gene expression levels

The reads were filtered according to Zhang et al. (2014) with minor modifications. The raw reads were filtered by removing read adaptors, artificial reads, and other low quality reads. After filtering, the clean reads were obtained and used for the subsequent analysis. The clean reads were mapped to the reference genome and assembled according to Lin et al. (2013) with some modifications. Using the programs TopHat 1.31 and Bowtie, the clean reads were mapped to the *A. flavus* genome, the EST sequencing and rRNA sequencing (Yu et al., 2004; Langmead et al., 2009; Trapnell et al., 2009). Using program Cufflinks, transcripts were assembled (Trapnell et al., 2010). The FPKM (Fragments Per Kb of exon per Million reads) method was used to calculate and normalize the expression levels of genes (Mortazavi et al., 2008).

### Identification and analysis of differentially expressed genes

The differentially expressed genes were identified and analyzed according to Lin et al. (2013) with minor modifications. The normalized gene expression levels in *A. flavus* treated with eugenol and untreated sample were directly compared. The *p*-value was then used to identify the differentially expressed genes. FDR (False Discovery Rate) control was used to correct *p*-value and FDR ≤ 0.05 was chosen. Finally, GO functional enrichment analysis and KEGG pathway enrichment analysis were performed using the FungiFun program (Kanehisa et al., 2008; Priebe et al., 2011).

### Reverse transcription (RT)-PCR conditions and q-PCR analysis of aflatoxin biosynthesis genes

First-strand cDNA was obtained by RT-PCR using the Takara RNA Kit (AMV) ver. 3.0 (Takara Bio Inc., Japan) according to the manufacturer's instructions. All PCR primers were designed based on the *A. flavus NRRL 3357* genomic sequence (GenBank accession number EQ963478A). Primers pair sequences of *18S, aflR, aflS (aflJ), aflA (fas-2), aflC (pksA), aflD (nor-1), aflE (norA), aflF (norB), aflG (avnA), aflH (adhA), aflI (avfA), aflJ (estA), aflK (vbs), aflL (verB), aflM (ver-1), aflN (verA), aflO (omtB), aflP (omtA), aflQ (ordA), aflU (cypA), aflX (ordB)*, and *aflT* were adapted from Liu et al. (2017) and the primer sequences of *aflB (fas-1), aflCa (hypC), aflLa (hypB), aflMa (hypE), aflNa (hypD), aflV (cypX), aflW (moxY)*, and *aflY (hypA)* were adapted from Caceres et al. (2016). All 29 genes encoding aflatoxin biosynthesis were analyzed. Real-time PCR was performed on an ABI Prism 7500 Sequence Detection System (Applied Biosystems, Foster City, CA, USA). MicroAmp optical 96-well plates were prepared for PCR with each well containing a total volume of 20 μL: 10 μL of SYBR Green Real-time PCR Master Mix (Applied Biosystems) used as the fluorescent dye, 2 μL of cDNA and 1 μL of each primer. The q-PCR steps were performed as previously described by Liu et al. (2017).

### Availability of RNA-seq data

The raw RNA-seq data of *A. flavus* discussed in this work have been deposited in the NCBI Sequence Read Archive under the accession number of SRP132641.

## Results

### RNA-seq data

RNA sequencing of eugenol treated and untreated *A. flavus* YC-15 generated a total of 16.99 Gb of valid data and 64.65 million read pairs (the average length is 150 bp). Of these, 53.87 million passed purity filtering standards, of which approximately 29.63 million (55.00%) were uniquely mapped to the genome of *A. flavu*s. Among all the 107.73 million reads, 64.21 million (59.60%) were mapped to the *A. flavus* genome and only 0.02% of reads were aligned to rRNA genes. The overall transcription levels of the genes were quantified with FPKM values. The results showed that 11,941 (88.55%) of the 13,485 gene models in the *A. flavus* genome database were expressed at least once in one of the six samples. In the control and eugenol-treated groups, 10,932 (81.07%) and 10,826 (80.28%) genes were expressed, respectively.

### Identification and functional analysis of differentially expressed genes

**Figure 1:**
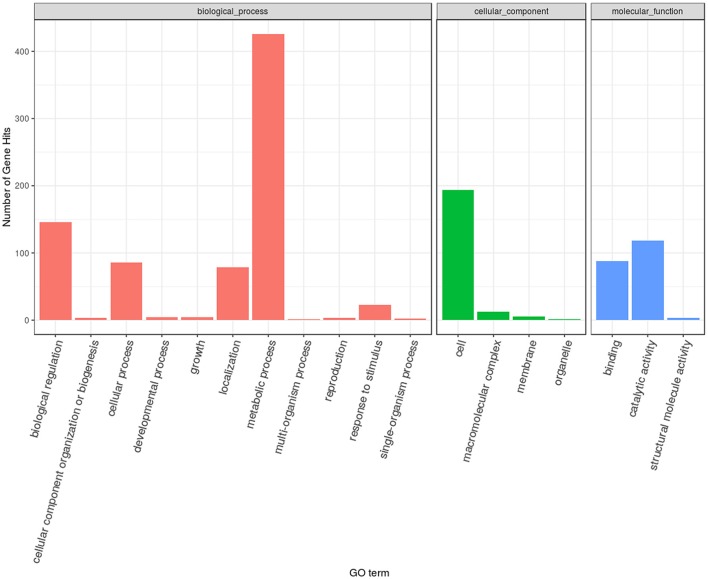
The gene ontology annotation of differential expression genes.

Based on the FPKM values, 735 differentially expressed genes were identified (with FDR ≤ 0.05, log_2_Ratio ≥ 1 or ≤1) between the eugenol and control groups. Among these, 271 gene displayed up-regulation and 464 genes displayed down-regulation after eugenol exposure. These differentially expressed genes were subjected to GO functional enrichment analysis. The results showed that these genes were mainly involved in RNA binding, hydrolase activity, pyrophosphatase activity, nucleoside-triphosphatase activity, structural molecular activity, transferase activity, methyltransferase activity, helicase activity, or macromolecular complex binding, or were structural constituents of the ribosome (Table 1, Figure 1). KEGG metabolic pathway enrichment analysis indicated that these genes were mainly involved in ribosome biogenesis, the ribosome, RNA transport, pyrimidine metabolism, and RNA polymerase (Table 2).

**Table 1:** GO functional enrichment analysis of differentially expressed genes when *A. flavus* was treated with eugenol.

**GO ID**	**TERM (Molecular function)**	***p-*Value**	***q-*Value**	**List hits**	**List size**
GO:0003723	RNA binding	2.56E-22	6.86E-20	99	800
GO:0016818	Hydrolase activity, acting on acid anhydrides	3.54E-03	3.38E-02	61	800
GO:0016462	Pyrophosphatase activity	4.87E-03	4.04E-02	60	800
GO:0017111	Nucleoside-triphosphatase activity	2.99E-03	2.96E-02	59	800
GO:0005198	Structural molecule activity	2.08E-03	2.42E-02	38	800
GO:0016741	Transferase activity, transferring one-carbon	2.75E-05	5.25E-04	36	800
GO:0008168	Methyltransferase activity	2.35E-05	4.83E-04	34	800
GO:0003735	Structural constituent of ribosome	1.78E-05	3.97E-04	33	800
GO:0004386	Helicase activity	7.41E-10	6.60E-08	29	800
GO:0044877	Macromolecular complex binding	4.76E-03	4.04E-02	25	800

**Table 2:** KEGG metabolic pathway enrichment analysis of differentially expressed genes when *A. flavus* was treated with eugenol.

**ID**	**TERM (Molecular function)**	***p-*Value**	***q-*Value**	**List hits**	**List size**
Afv03008	Ribosome biogenesis in eukaryotes	1.63E-16	1.39E-14	36	313
Afv03010	Ribosome	7.74E-09	3.30E-07	26	313
Afv03013	RNA transport	1.44E-03	2.45E-02	23	313
Afv00240	Pyrimidine metabolism	9.68E-04	2.06E-02	17	313
Afv03020	RNA polymerase	1.86E-04	5.30E-03	11	313

### Analysis of gene expression in the pigment, aflatrem, aflatoxin, and cyclopiazonic acid secondary metabolite pathways in *A. flavus* treated with eugenol

Using the SMURF program and the website of the Center for Integrated Fungal Research (Lin et al., 2013), 55 secondary metabolite pathways of *A. flavus* were identified and analyzed. The transcription levels in most of these pathways were not significantly affected by eugenol. The transcription levels of the genes involved in the biosynthesis of conidial pigment (#10), aflatrem (#15), aflatoxin (#54), and cyclopiazonic acid (#55) are showed in Table 3. In pathway #10, AFLA_016120 encoding an O-methyltransferase family protein and AFLA_016130 were down-regulated. In pathway #15, most of cluster genes were expressed at a very low level. In pathway #55, AFLA_139490 encoding a hybrid PKS/NRPS enzyme and AFLA_139470 encoding a FAD dependent oxidoreductase were up-regulated, whereas AFLA_139460 encoding a MFS multidrug transporter was down-regulated. In previous studies, we found that aflatoxin biosynthesis was repressed in *A. flavus* treated with eugenol (Liang et al., 2015). However, in the present study, most genes in pathway #54 were expressed at high levels with only slight changes after eugenol exposure. Of 29 genes in the aflatoxin biosynthetic cluster, 19 genes' transcription were down-regulated to varying degrees, including the key structural genes *aflI, aflJ, aflH, aflE, aflG, aflM, aflD, aflP*, and *afL* (Table 3). Most surprising of all, the expression of the regulator gene *aflR* did not change significantly and the expression of *aflS* was slightly up-regulated (Table 3).

**Table 3:** Transcriptional activity of genes in the biosynthesis of conidial pigment (#10), aflatrem (#15), aflatoxin (#54), and cyclopiazonic acid (#55).

**Cluster ID**	**Gene ID (AFLA_x)**	**Untreated (FPKM)**	**D125 (FPKM)**	**Log**	**Annotated_gene_function**
#10	016120	10.58	3.02	−1.81	O-methyltransferase family protein
#10	016130	13.25	6.45	−1.04	Hypothetical protein
#10	016140	14.10	17.20	0.29	Conidial pigment biosynthesis scytalone dehydratase Arp1
#15	045450	37.27	64.22	0.79	Ankyrin repeat-containing protein, putative
#15	045460	1.16	5.26	2.18	Hypothetical protein
#15	045470	0.10	0	/	Nonsense-mediated mRNA decay protein, putative
#15	045480	0.32	1.27	2.00	Conserved hypothetical protein
#15	045490	0.03	0.16	2.18	Dimethylallyl tryptophan synthase, putative
#15	045500	0.55	0.57	0.05	Cytochrome P450, putative
#15	045510	0.12	0	/	Integral membrane protein
#15	045520	0	0	/	Integral membrane protein
#15	045530	0.23	0	/	Conserved hypothetical protein
#15	045540	0	0.06	/	Cytochrome P450, putative
#15	045550	1.24	1.12	−0.14	Hypothetical protein AFLA_045550
#15	045560	1.94	1.89	−0.04	Carboxylic acid transport protein
#15	045570	1.55	4.59	1.57	Acetyl xylan esterase, putative
#54	139100	2.96	2.43	−0.28	*aflYe/orf*/Ser -Thr protein phosphatase family protein
#54	139110	2.38	2.74	0.20	*aflYd*/sugR/sugar regulator
#54	139120	1.85	1.93	0.06	*aflYc/glcA*/glucosidase
#54	139130	1.78	2.49	0.49	*aflYb*/hxtA/putative hexose transporter
#54	139140	5.53	7.09	0.36	*aflYa/*nadA/NADH oxidase
#54	139150	101.03	92.37	−0.13	*aflY/hypA/hypP*/hypothetical protein
#54	139160	117.97	96.36	−0.29	*AflX/ordB*/monooxygenase
#54	139170	49.07	43.95	−0.16	*aflW/moxY*/monooxygenase/oxidase
#54	139180	48.91	53.16	0.12	*aflV/cypX*/cytochrome P450 monooxygenase
#54	139190	112.43	140.97	0.33	*aflK/vbs*/VERB synthase
#54	139200	12.91	14.38	0.16	*aflQ/ordA/ord-1*/oxidoreductase/cytochrome P450 monooxigenase
#54	139210	92.70	72.78	−0.35	*aflP/omtA/omt-1*/O-methyltransferase A
#54	139220	187.22	164.37	−0.19	*aflO/omtB/dmtA*/O-methyltransferase B
#54	139230	15.55	9.20	−0.76	*aflI/avfA*/cytochrome P450 monooxygenase
#54	139240	108.16	87.23	−0.31	*aflLa/hypB*/hypothetical protein
#54	139250	92.87	75.90	−0.29	*aflL/verB*/desaturase/P450 monooxygenase
#54	139260	48.69	35.41	−0.46	*aflG/avnA/ord-1*/cytochrome P450 monooxygenase
#54	139270	572.31	386.33	−0.57	*aflNa/hypD*/hypothetical protein
#54	139280	34.04	34.80	0.03	*aflN/verA/*monooxygenase
#54	139290	136.29	75.22	−0.86	*aflMa/hypE*/hypothetical protein
#54	139300	496.53	375.07	−0.40	*aflM/ver-1*/dehydrogenase/ketoreductase
#54	139310	180.51	122.95	−0.55	*aflE/norA/aad/adh-2*/NOR reductase
#54	139320	132.91	78.55	−0.76	*aflJ/estA/*esterase
#54	139330	192.79	128.47	−0.59	a*flH/adhA*/short chain alcohol dehydrogenase
#54	139340	177.63	200.51	0.17	*aflS/*pathway regulator
#54	139360	64.90	69.27	0.09	*aflR/apa-2/afl-2*/transcription activator
#54	139370	35.31	38.25	0.12	*aflB/fas-1*/fatty acid synthase beta subunit
#54	139380	19.45	17.79	−0.13	*aflA/fas-2/hexA*/fatty acid synthase alpha subunit
#54	139390	231.56	180.60	−0.36	*aflD/nor-1*/reductase
#54	139400	84.14	51.61	−0.71	*aflCa/hypC/*hypothetical protein
#54	139410	37.55	38.39	0.03	*aflC/pksA/pksL1*/polyketide synthase
#54	139420	100.86	99.84	−0.01	*aflT/aflT*/transmembrane protein
#54	139430	20.76	26.04	0.33	*aflU/cypa*/P450 monooxygenase
#54	139440	14.48	15.48	0.10	*aflF/norB*/dehydrogenase
#55	139460	1293.63	1017.37	−0.35	MFS multidrug transporter, putative
#55	139470	215.54	296.72	0.46	FAD dependent oxidoreductase, putative
#55	139480	243.62	278.43	0.19	tryptophan dimethylallyltransferase
#55	139490	9.14	17.52	0.94	Hybrid PKS/NRPS enzyme, putative

### Confirmation analysis of gene expression involved in aflatoxin biosynthesis

**Figure 2:**
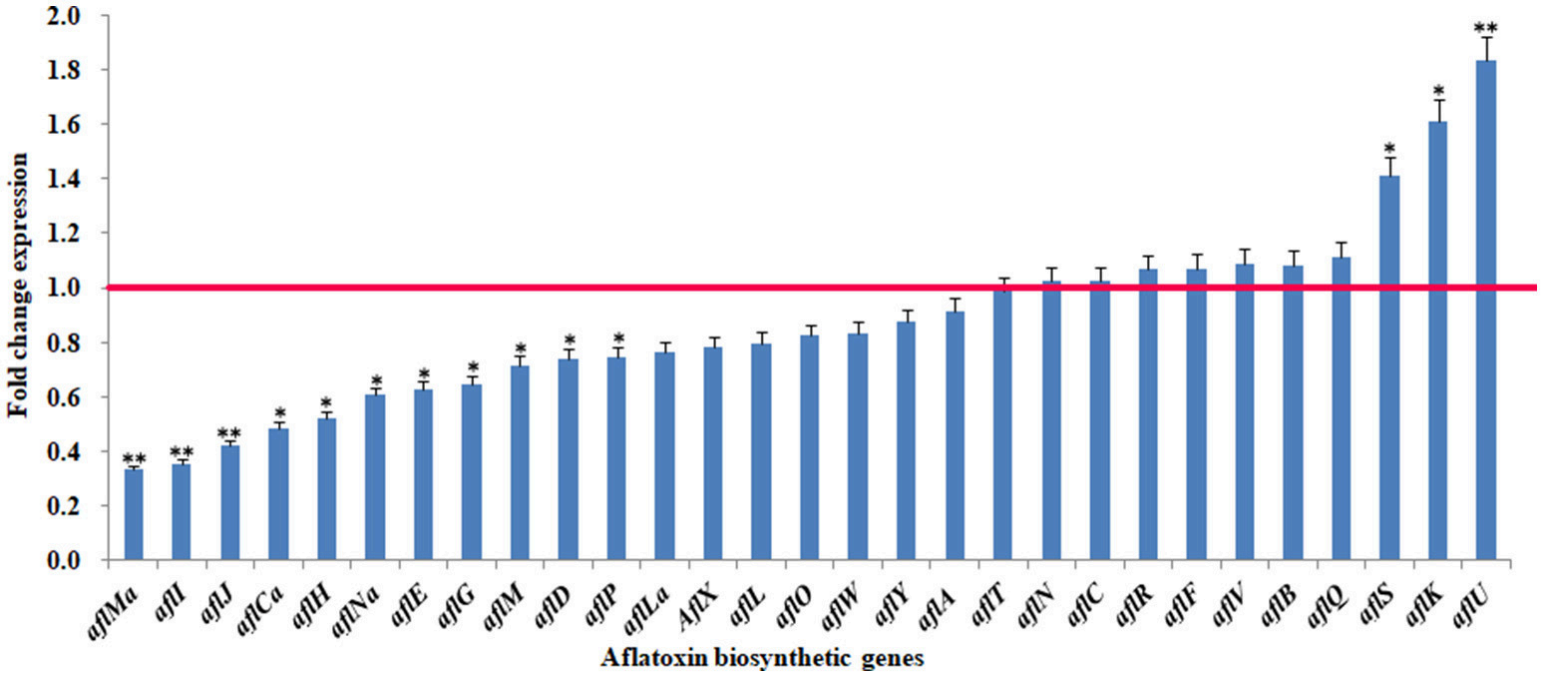
Fold change expression of genes belonging to the cluster responsible for aflatoxin biosynthesis in response to eugenol at 0.80 mM. Red line represents control expression level. *p-value < 0.05; **p-value < 0.01.

To confirm the observed changes in aflatoxin biosynthetic gene expression, two regulator genes (*aflS* and *aflR*) and all the structural genes (*aflI, aflJ, aflH, aflE*, etc.) were further analyzed by quantitative real-time PCR (q-PCR). The results showed that the transcription of 19 genes was down-regulated to varying degrees after eugenol exposure (Figure 2), which was consistent with the RNA-seq data. For example, *aflMa* was the most strongly down-regulated gene in *A. flavus* treated with eugenol in both the RNA-Seq and q-PCR analyses.

### Analysis of fungal development-related gene expression in *A. flavus* treated with eugenol

From the gene expression pattern data, we found that the expression levels of some genes involved in conidiophore development were dysregulated after exposure to eugenol (Table 4). The *veA* gene (AFLA_066460), encoding a global regulator, was down-regulated with its FPKM value decreasing from 312.87 to 204.64. The transcription of the conidia-specific hydrophobin gene *RodA* (AFLA_098380) was down-regulated with its FPKM decreasing from 12.35 to 5.42. However, the transcription of the conidial hydrophobin gene *RodB* (AFLA_014260) was up-regulated with its FPKM increasing from 2.51 to 17.48. The transcription of the C_2_H_2_ type conidiation transcription factor *BrlA* (AFLA_082850) was up-regulated with it FPKM increasing from 2.00 to 7.08. In addition, the transcription levels of the transcription factor *AbaA* (AFLA_029620), the development regulator *FlbA* (AFLA_134030), the APSES transcription factor *StuA* (AFLA_046990), and the transcription factor *Medusa* (AFLA_136410) showed a mild up-regulation.

**Table 4:** Transcriptional activity of genes involved in *A. flavus* development.

**Gene ID (AFLA_x)**	**Untreated (FPKM)**	**D125 (FPKM)**	**Log**	**Annotated_gene_function**
066460	312.87	204.64	−0.61	Developmental regulator *AflYf/VeA*
033290	31.74	29.12	−0.12	Regulator of secondary metabolism *LaeA*
014260	2.51	17.48	2.80	Conidial hydrophobin *RodB/HypB*
098380	12.35	5.42	−1.19	Conidial hydrophobin *RodA/RolA*
081490	36.78	23.90	−0.62	Nucleoside diphosphatase *Gda*1
018340	46.12	54.38	0.21	G-protein complex alpha subunit *GpaA/FadA*
046990	148.93	234.07	0.65	APSES transcription factor *StuA*
136410	89.17	135.16	0.60	Transcriptional regulator *Medusa*
029620	1.02	1.69	0.73	Transcription factor *AbaA*
082850	2.00	7.08	1.82	C_2_H_2_ type conidiation transcription factor *BrlA*
039530	9.85	11.78	0.26	FluG family protein
071090	893.92	758.74	−0.24	GPT-binding protein *EsdC*
101920	2.17	3.35	0.63	Extracellular developmental signal biosynthesis protein FluG
020210	86.15	85.76	−0.01	Sexual development transcription factor *NsdD*
026900	10.56	12.68	0.26	Developmental regulator *VosA*
052030	7.22	8.03	0.15	Developmental regulatory protein *WetA*
131490	37.64	41.06	0.13	Conserved hypothetical protein
134030	10.32	16.26	0.66	Development regulator *FlbA*
137320	104.86	92.80	−0.18	C_2_H_2_ conidiation transcription factor *FlbC*

### Analysis of the expression of genes involved in fungal oxidative stress in *A. flavus* treated with eugenol

The transcription levels of oxidative stress-related genes are shown in Table 5. Among the 47 relevant genes, 23 genes were significantly modulated by eugenol. The expression of dioxygenase-encoding *ppoC*, the transcriptions factor genes *msnA, srrA* and *pacC*, the cellular receptor *gprC, gprF, gprK, gprM*, and *gprS*, the MAP kinase genes *bck1, ste11*, and *sskB* and the superoxide dismutase gene *sod1* were up-regulated. Conversely, the addition of eugenol induced the down-regulation of 10 genes encoding for (i) oxylipins *ppoB* (ii) GPCRs (*gprA, gprD, gprG, gprO*, and *gprB*) (iii) protein kinase (*sakA* and *maf1*) (iiii) catalase *cat2* (iiiii) dehydrogenase *gfdB*.

**Table 5:** Transcriptional activity of MAPK pathway, Oxylipins, and GPCRs genes in *A. flavus*.

**Gene ID (AFLA_x)**	**Gene**	**Untreated (FPKM)**	**D125 (FPKM)**	**Log**	**Annotated_gene_function**
062500	*Maf1*	66.38	51.64	−0.36	Mitogen-activated protein kinase
083380	*Pbs2*	35.91	39.53	0.14	MAP kinase kinase
103480	*Ste7*	9.60	11.75	0.29	MAP kinase kinase
048880	*Ste11*	13.04	16.99	0.38	MAP kinase kinase kinase
035530	*Ste20*	52.79	52.11	−0.02	Serine/threonine kinase
021030	*/*	25.45	23.47	−0.12	Serine/threonine protein kinase
052570	*mpkA*	44.75	43.77	−0.03	MAP kinase
051240	*Mkk2*	100.19	108.85	0.12	MAP kinase kinase
034170	*Fus3*	85.07	89.43	0.07	MAP kinase
031560	*bck1*	12.93	19.95	0.63	MAP kinase kinase kinase
100250	*cat*	0.40	0.38	−0.07	Catalase Cat
090690	*Cat1*	180.19	167.61	−0.10	Mycelial catalase
122110	*Cat2*	20.23	12.20	−0.73	Bifunctional catalase-peroxidase
056170	*catA*	612.92	648.71	0.08	Spore-specific catalase
099000	*sod1*	98.10	125.45	0.35	Cu, Zn superoxide dismutase SOD1
033420	*mnSOD*	1175.47	1008.01	−0.22	Mn superoxide dismutase
031340	*atfA*	227.96	214.80	−0.09	bZIP transcription factor
094010	*atfB*	212.65	230.11	0.11	bZIP transcription factor
129340	*ap-1*	158.11	135.16	−0.23	bZIP transcription factor AP-1
110650	*msnA*	80.07	188.50	1.24	C_2_H_2_ transcription factor
091490	*mtfA*	34.54	28.89	−0.26	C_2_H_2_ finger domain protein
030580	*pacC*	129.52	165.07	0.35	C_2_H_2_ transcription factor
034540	*srrA*	45.97	72.01	0.65	Stress response transcription factor
062210	*sskA*	22.66	23.11	0.03	Response regulator
068590	*sskB*	14.76	18.52	0.33	MAP kinase kinase kinase
061090	*sakA*	2.05	1.23	−0.74	MAP kinase
026790	*ppoA*	22.17	26.58	0.26	Fatty acid oxygenase
120760	*ppoB*	0.49	0.03	−4.03	Fatty acid oxygenase
030430	*ppoC*	1.04	5.19	2.32	Fatty acid oxygenase
002850	*AfPXG*	395.51	335.03	−0.24	Calcium binding protein Caleosin/Peroxygenase
025100	*gpdA*	7357.45	7758.80	0.08	Glyceraldehyde 3-phosphate dehydrogenase
046760	*gfdB*	320.94	253.48	−0.34	Glycerol 3-phosphate dehydrogenase
060740	*gprA*	11.82	5.36	−1.14	STE3 GPCR (*S. cerevisiae* pheromone receptor)
061620	*gprB*	7.64	6.13	−0.32	STE3 GPCR (*S. cerevisiae* pheromone receptor)
074150	*gprC*	2.56	8.76	1.77	Git3; Git3_C (*S. pombe* glucose sensor)
135680	*gprD*	10.67	5.34	−1.00	Git3; Git3_C (*S. pombe* glucose sensor)
006880	*gprF*	45.33	68.78	0.60	PQ loop repeat (*S. pombe* nitrogen sensor)
067770	*gprG*	40.30	22.59	−0.84	PQ loop repeat (*S. pombe* nitrogen sensor)
006920	*gprH*	0.78	0.72	−0.12	Secretin family (signal through cAMP pathways)
127870	*gprJ*	50.86	61.23	0.27	Vacuolar membrane PQ loop repeat protein
009790	*gprK*	0.25	0.42	0.75	RGS domain (regulator of G protein signaling)
075000	*gprM*	3.33	5.43	0.71	Conserved hypothetical protein
032130	*gprO*	40.06	27.13	−0.56	Hemolysin III related (broad range of ligands)
088190	*gprP*	33.21	31.68	−0.07	Hemolysin III related (broad range of ligands)
023070	*gprR*	32.65	30.65	−0.09	RGS domain (regulator of G protein signaling)
006320	*gprS*	12.95	16.26	0.33	PQ loop repeat protein
117970	*nopA*	5388.07	4310.21	−0.32	Bacteriorhodopsin-like (photoreactive)

## Discussion

To guarantee the health of human beings and food safety, chemical fungicides have been gradually limited in the food chain. In recent years, a number of essential oils from plants have been widely used in food industries as alternatives to chemical fungicides. The inhibitory effects of essential oils, such as citral, cinnamon, clove, litsea, eucalyptus, ginger, anise, spearmint, and camphor oils on fungal growth and toxin production have been reported by many researchers (Velluti et al., 2003; Liang et al., 2015). In our earlier study, of these natural plant-based compounds, 0.80 mM eugenol significantly reduced AFB_1_ production with inhibitory rate 95.4%, but with no effect on fungal growth (Liang et al., 2015). In this study, the mechanism by which eugenol dysregulates *A. flavus* growth and aflatoxin production was studied using an RNA-seq analysis.

The expression of 19 of 29 genes in the aflatoxin biosynthetic pathway cluster was down-regulated when *A. flavus* was treated with eugenol. However, the expression of none of these genes was completely inhibited. The most strongly down-regulated gene was *aflMa*, followed by *aflI, aflJ, aflCa, aflH, aflNa, aflE, aflG, aflM, aflD, aflP*, and *aflLa*. These observed changes in aflatoxin biosynthetic gene transcript levels were confirmed with q-PCR. *AflD (nor-1)* and *aflE* (*norA*) both encode reductases that are involved in the conversion of NOR (norsolorinic acid) to AVN (averantin). *AflG* (*avnA*), encoding a cytochrome P450 monooxygenase, converts AVN to HAVN (5′-hydroxy-averantin). *AflH* (*adhA*) encodes an alcohol dehydrogenase which is involved in the conversion of HAVN to AVF (averufin). *AflI* (*avfA*) encodes an oxidase that converts AVF to VHA (versiconal hemiacetal acetate). *AflJ* (*estA*) encodes an esterase that is necessary for the conversion of VHA to VAL (versiconal) (Yu et al., 2004; Cleveland et al., 2009). Our results indicated that the pathway from NOR to VAL was repressed in *A. flavus* treated with eugenol. *AflL* (*verB*) encodes a P450 monooxygenase/desaturase which converts VERB (versicolorin B) to VERA (versicolorin A). *AflM* (*ver-1*) encodes a dehydrogenase that can convert VERA to DMST (demethylsterigmatocystin). *AflO* (*omtB*) encodes an O-methyltransferase and converts DMST to ST (sterigmatocystin). *AflP* (*omtA*) encodes another O-methyltransferase and converts ST to OMST (O-methylsterigmatocystin) (Yu et al., 2004). In the present study, the transcription of *aflL, aflM, aflO*, and *aflP* was all down-regulated by eugenol, suggesting that the aflatoxin biosynthetic pathway from VERB to OMST was also repressed in *A. flavus* treated with eugenol. We obtained similar results in a previous study with q-PCR, and found that the transcription of *aflP, aflM*, and *aflD* was also down-regulated by 0.80 mM eugenol (Liang et al., 2015). *AflMa (hypE*) encodes an enzyme (HypE) which, together with AflE, may be involved in the final two steps in aflatoxin biosynthesis. *AflCa* (*hypC*) encodes an oxidase which catalyzes the oxidation of norsolorinic acid anthrone. *AflNa* (*hypD*) encodes an integral membrane protein that inhibits *A. flavus* growth and aflatoxin biosynthesis. *AflLa* (*hypB*) encodes an oxidase that is assumed to be involved in one of the oxidation steps in the conversion of OMST to aflatoxin (Wei et al., 2014). These results suggested that eugenol inhibited aflatoxin biosynthesis in *A. flavus* treated with eugenol by down-regulating the expression of several structural genes.

Most surprising of all, the transcription regulator gene *aflR* in the cluster did not show significant differential expression after treatment with eugenol, while the transcription regulator gene *aflS* showed a slight up-regulation (Table 3). The results are similar with the findings of Lin et al. (2013). They found that the transcription regulator genes *aflR* and *aflS* showed no significant differential expression after treatment with 5-Azacytidine (5-Ac), an inhibitor of aflatoxin production and development in *A. flavus*. However, the expressions of *aflQ, aflI*, and *aflLa* were totally or almost totally inhibited by 5-Ac. Aflatoxins are produced optimally at 28–30°C and production significantly decreases as temperature approach 37°C, the optimum temperature for fungal growth. OBrian et al. (2007) found that transcript levels of *aflR* and *aflS* did not change significantly between 28 and 37°C, while all the structural genes were much lowly expressed at 37°C relative to 28°C. *A. flavus* exhibits decreased conidiation and aflatoxin biosynthesis under water activity (*a*_w_) 0.93 compared to that under 0.99 *a*_w_. Zhang et al. (2014) found that transcript levels of *aflR* and *aflS* both showed no significant differential expression between two water activities using RNA-seq approach, while the expression of 16 aflatoxin producing-related genes decreased obviously when *a*_w_ decreased. There are five potential explanations for the decreased transcription of several aflatoxin biosynthesis structural genes while the *aflR* did not change significantly and *aflS* was slightly up-regulated after treatment with eugenol: (a) some other transcription regulators may be involved in the down-regulating of these structural genes; (b) post-transcriptional regulation influences the expression of these structural genes; (c) less AFLR is produced after treatment with eugenol and translation process may be involved in the modulation of these structural genes; (d) AFLR is nonfunctional with eugenol exposure; (e) AFLR and AFLS are unable to interact with eugenol exposure.

RNA-binding proteins, which binding to the double or single stranded RNA in cells, participate in the formation of ribonucleoprotein complexes. However, most RNA-binding proteins exist as complexes of protein and pre-mRNA called heterogeneous ribonucleoprotein particles (hnRNPs) because most mature RNA is exported rapidly from the nucleus. RNA-binding proteins have crucial roles in various cellular processes, including cellular function, transport and localization. In particular, they play a major role in the post-transcriptional regulation of RNAs, including their polyadenylation, splicing, mRNA stabilization, localization and translation (Glisovic et al., 2008). Bai et al. (2015) investigated the effects of temperature on transcripts and the corresponding proteins levels using transcriptome-proteome correlation analysis and found that the correlation between protein concentrations and transcript levels was low in *A. flavus*. Therefore, they proposed that the post-transcriptional regulation process may be involved in aflatoxin biosynthesis (Bai et al., 2015). In the present study, GO functional enrichment analysis showed that RNA binding was the most dysregulated function in *A. flavus* treated with eugenol, suggesting that post-transcriptional regulation process may be involved in the inhibition of aflatoxin biosynthesis by eugenol.

Ribosome biogenesis is the process by which ribosomes are constructed. In eukaryotes, it takes place in both cytoplasm and the nucleolus. Ribosome biogenesis is intimately associated with many cellular activities including growth, division and secondary metabolism and its process is very tightly regulated (Thomson et al., 2013). The vesicle-vacuole was involved in the conversion of sterigmatocystin (ST) to aflatoxin B_1_ and compartmentalizing of aflatoxin in *A. parasiticus* (Chanda et al., 2009). The free ribosomes in the cytoplasm are the sites where the three key enzymes Nor-1, Ver-1, and OmtA are synthesized (Chanda et al., 2009). After synthesis, these three enzymes are packaged into transport vesicles and transported to vacuoles *via* the cytoplasm-to-vacuole targeting pathway (Chanda et al., 2009). Beside the above pathway, the latest research found that the aflatoxin biosynthesis, exporting, and secretion also occur *via* cytoplasmic lipid droplets and their associated proteins, oleosins and caleosins (Hanano et al., 2018). Therefore, the free ribosomes also play a critical role in the aflatoxin biosynthesis. In the present study, KEGG metabolic pathway enrichment analysis indicated that ribosome biogenesis was the most dysregulated metabolic pathway in *A. flavus* treated with eugenol. The result suggested that eugenol dysregulated ribosome biogenesis, which then prevented the synthesis of Nor-1, Ver-1, and OmtA, and the subsequent formation of aflatoxin.

The excess reactive oxygen species (ROS) induces damages to DNA, proteins or lipids, and then causes alteration of cellular functions (Montibus et al., 2013). In *Aspergilli*, the stress signal transduction is activated by G Protein-Coupled Receptors (GPCRs) and oxylipins (Caceres et al., 2017). These cellular receptors also play an important role in secondary metabolite production (Yu and Keller, 2005). Several compounds inhibit the biosynthesis of aflatoxin in fungi by reducing the ROS levels via the activation of the antioxidant system (Reverberi et al., 2005; Grintzalis et al., 2014; Sun et al., 2015). The transcript levels of oxidative stress-related genes were presented in Table 5. After the addition of eugenol, 10 GPCRs, and two oxylipins genes were significantly regulated. Affeldt et al. (2014) reported that *gprK* deletion resulted in more aflatoxin in *A. flavus* treated with inhibitor methyl jasmonte. In this study, the up-regulation of *gprK* expression was associated with the reduction of AFB_1_ after eugenol exposure. Caceres et al. (2017) also found that over expressed *gprK* was associated with AFB_1_ inhibition by piperine. Oxylipins pathway are known to play important roles in aflatoxin biosynthesis, exporting, fungal development and seed infection. The recent publications show that the oxylipins pathway includes four genes, *ppoA, ppoB, ppoC*, and *afPXG*, in *A. flavus* (Tsitsigiannis and Keller, 2007; Affeldt et al., 2012; Hanano et al., 2015, 2018). AfPXG, the *A. flavus* caleosin with peroxygenase activity, is associated with the membrane of lipid droplets and mediates fungal development, aflatoxin accumulation, secretion, and seed infection (Hanano et al., 2015, 2018). Among the oxylipins genes, *ppoB* was the most impacted gene after eugenol exposure in this study. The deletion of *ppoB* induced more ST which is a precursor of AFB_1_ (Tsitsigiannis and Keller, 2006), implying a negative correlation between the up-regulation of *ppoB* and aflatoxin biosynthesis. Caceres et al. (2017) also found that the over expressed *ppoB* levels was associated with AFB_1_ inhibition by piperine. However, in the present study, reduced *ppoB* expression was associated with AFB_1_ inhibition by eugenol. All these results suggest that the function of GPCRs and oxylipins in aflatoxin production is complicated.

VeA, a global regulator, bridges VelB, and LaeA to form the velvet complex regulating fungal development and secondary metabolism such as aflatoxin (Lin et al., 2013). In addition, it is also involved in the oxidative stress response in *A. flavus* because it modulates the high osmolarity glycerol (HOG) signaling pathway genes (Duran et al., 2007; Caceres et al., 2017). In *A. flavus, veA* deletion resulted in the complete inhibition of *aflR, aflD, aflM*, and *aflP* expression, and the consequent absence of aflatoxin (Duran et al., 2007). In this study, the decreased expression of *veA* was also associated with reduced *aflM, aflD*, and *aflP* expression and the consequent reduction of aflatoxin production when eugenol treatment. The deletion of *veA* induced the down-regulation of oxidative stress-related genes such as *srrA, msnA*, and *atfA* (Baidya et al., 2014). However, *msnA* and *srrA* were up-regulated by eugenol in the present study. These results imply that other regulator factors are also involved in the anti-aflatoxigenic mechanism of eugenol.

In *A. parasiticus*, the bZIP transcription factors SrrA, AtfB, MsnA, and AP-1 were demonstrated as co-regulators of aflatoxin biosynthesis and oxidative stress (Hong et al., 2013; Caceres et al., 2017). In the present study, we found that the genes encoding these proteins play important roles in the anti-aflatoxigenic mechanism of eugenol. Among these genes, the *msnA* gene was the most highly up-regulated gene by eugenol. *MsnA*, encoding a C_2_H_2_-type zinc-finger regulator, plays a critical role in fungal growth, aflatoxin and kojic acid biosynthesis, and the oxidative stress response. In *A. flavus* and *A. parasiciticus, msnA* deletion resulted in retarded colony growth, slightly increased production of aflatoxin and elevated the production of kojic acid (Chang et al., 2010). In this study, a good correlation between the up-regulation of *msnA* and the decrease of aflatoxin in *A. flavus* treated with eugenol. This confirmed that the transcription factor MsnA down-regulated the production of AFB_1_. Caceres et al. (2016) also found that the *msnA* gene was up-regulated by 1.9 times after eugenol exposure using a large-scale q-PCR approach. In the present study, *atfB* and *srrA* were also up-regulated by eugenol. However, *ap-1* was down-regulated. These results suggested that the increased transcription level of the bZIP transcription factor genes *msnA, atfB*, and *srrA* is directly involved in the reduced production of aflatoxin induced by eugnol.

The antioxidant-related genes such as genes encoding superoxide dismutases (SODs) and catalase (CAT), are also modulated by the bZIP transcription factors and are involved in the cellular defense against oxidative stress (Caceres et al., 2017). In the present study, the expression of *sod1* was up-regulated while *cat2* and *mnSOD* were down-regulated. Many publications have reported that several inhibitors can inhibit aflatoxin production by modulating the antioxidant activities of the fungus (Caceres et al., 2017). However, the effect of inhibitors on the enzymatic defense depends on the type of aflatoxin inhibitor. For example, dithiothreitol, dimethyl sulfoxide, and β-glucans from *Lentinula edodes* resulted in AFB_1_ decrease accompanied with a rising in CAT activity (Reverberi et al., 2005; Grintzalis et al., 2014; Caceres et al., 2017). Conversely, ascorbic acid and cinnamaldehyde greatly reduced the production of AFB_1_ with a rising in SOD activity (Grintzalis et al., 2014; Sun et al., 2015; Caceres et al., 2017). These results indicate that eugenol promotes SOD activity as part of the mechanism of action occurring during AFB_1_ inhibition.

As a global regulator, VeA is also a critical element coordinating fungal development. VeA trans-regulates the expression of *brlA* gene which encodes an early regulator of fungal development by modulating the α/β transcript ratio (Kato et al., 2003; Lin et al., 2013). Interestingly, the *brlA* gene was evidently up-regulated by eugenol in the present study (Table 4). Then the over-expression of *brlA* gene will activate fungal conidiation and growth. Similarly, Lin et al. (2013) found that *brlA* gene was up-regulated in *A. flavus* treated with 5-Azacytidine (5-AC). Calvo et al. (2004) found that *brlA* gene was highly expressed in the *veA* deletion strain of *A. nidulans*. The activation *brlA* is an essential step of conidiation in *Aspergillus* (Adams et al., 1988). The BrlA protein includes two C_2_H_2_ zinc finger motifs and controls early developmental regulatory genes including *abaA, rodA*, and *yA* (Clutterbuck, 1969; Boylan et al., 1987; Chang and Timberlake, 1993; Timberlake and Clutterbuck, 1994). BrlA activates AbaA which plays a critical role in proper differentiation and action of phialides (Sewall et al., 1990; Andrianopoulos and Timberlake, 1994). In this study, the transcription of *brlA* in *A. flavus* treated with eugenol was up-regulated, resulting in the up-regulation of *abaA* and subsequent activation of fungal conidiation and development.

The *flbA* gene, encoding a development regulator, plays an important role in the expression of *nsdD* and *esdC* (Han et al., 2001, 2008). The expression of *nsdD* and *esdC* was inhibited by activating FadA and SfaD, directly or indirectly (Han et al., 2008). In this study, the expression of *flbA* and *fadA* gene was up-regulated whereas that of *esdC* gene was slightly down-regulated. Taken together, *brlA* and *flbA* were up-regulated by the repression of the *veA* gene in *A. flavus* treated with eugenol. The increased FlbA activated FadA and SfaD. Therefore, activated FlbA induced asexual development and sexual development through the up-regulation of *brlA* gene and the down-regulation of *esdC* gene, respectively.

Eugenol can be biotransformed by some microbial enzymes. A few eugenol-converting enzymes have been reported, including vanillyl-alcohol oxidase from *Penicillium simplicissimum* (de Jong et al., 1992), 4-thylphenol methylenehydroxylase from *Pseudomonas putida* DJ1 (Reeve et al., 1989), eugenol dehydrogenase from *Pseudomonas fluorescens* E118 (Furukawa et al., 1998), laccase from fungi (Qi et al., 2015), eugenol oxidase from *Rhodococcus* sp. strain RHA1 (Jin et al., 2007), and lipase from *Candida antarctica* and *Staphylococcus aureus* (Horchani et al., 2010; Chiaradia et al., 2012). In *A. flavus*, there are many similar enzyme genes as well as, such as alcohol dehydrogenase (AFLA_004360, AFLA_005070, AFLA_008880, AFLA_010050, AFLA_024270, AFLA_024700, AFLA_038770, and AFLA_128700), laccase (AFLA_000890 and AFLA_123160), and lipase (AFLA_016150, AFLA_020170, AFLA_057690, and AFLA_058010). In this study, these genes were up-regulated in *A. flavus* treated with eugenol (Table S2). The results mean that eugenol may be converted by *A. flavus*. Our previous study showed *aflD* and *aflM* were up-regulated at 6–7 d while they were down-regulated at 1–5 d, suggesting that eugenol might be converted to other compounds having lower antiaflatoxigenic activities (Liang et al., 2015).

**Figure 3:**
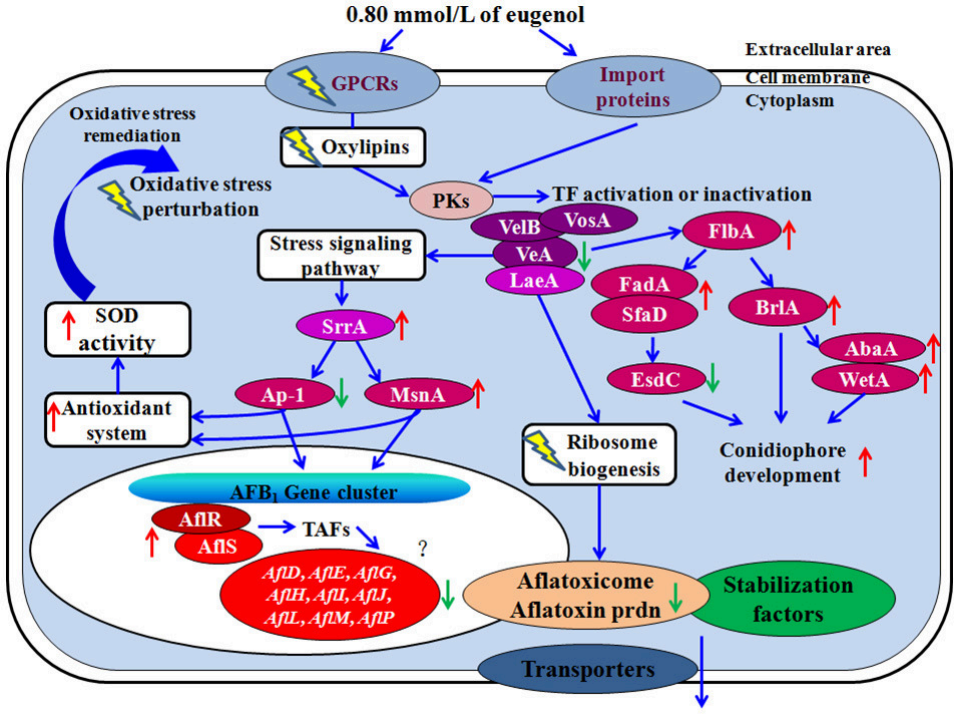
Hypothetical mechanism of action of eugenol. Eugenol perturbs cellular signaling pathway by modulating GPCRs and oxylipins expression levels. Simultaneously, decreased levels of *veA* might make fungus less tolerant to oxidative stress response which could trigger an activation of several genes involved in the stress signaling pathway such as stress response transcription factor *srrA*, C_2_H_2_ transcription factor *msnA*, and down-regulate bZIP transcription factor *ap-1*. Final targets of these modulators correspond to fugal antioxidant system consisting in genes coding for catalasess and superoxide dismutase defenses. The down regulation of genes belonging to the AFB_1_ cluster may then be a final sequence of the repressive modulation caused by the over expression of OSR transcription factors. Dysregulation of ribosome biogenesis prevents the biosynthesis of Nor-1, Ver-1, and OmtA, and the aflatoxisomes from performing their normal function in aflatoxin formation. For conidiophores development, the growth signaling from the activated *fadA* and *sfaD* should be improved by *flbA*. Upon the activation of them, *flbA* causes asexual development by the activation of the *brlA* gene and repression of *esdC* gene. Up- or down-regulation of gene upon eugenol addition is represented by red and green arrow. PKs, protein kenase; TF, transcription factor.

To decipher the molecular mechanism of action on the aflatoxin production and fungal growth in *A. flavus* treated with eugenol, we proposed a hypothetical gene modulation mode of action (Figure 3).

## Conclusions

In the present study, we have proposed a mechanism to explain the transcription modulation behind the inhibitory function of eugenol on AFB_1_ using an RNA-seq analysis. Based on the results in previous publications and this study, we concluded that (i) the reduction of aflatoxin biosynthesis is due to the down-regulation of most aflatoxin pathway structural genes in *A. flavus* treated with eugenol, (ii) eugenol's transcription modulation mechanism includes the down-regulation of the global regulator *veA* accompanied with the up-regulation of the oxidative stress-related transcription factor genes *msnA* and s*rrA*, and down-regulation of *ap-1* and *mtfA*, (iii) eugenol induces dysregulated transcription of GPCRs and oxylipins genes, (iiii) post-transcription modification and backbone enzymes biosynthesis may be involved in the inhibition of AFB_1_ production by eugenol.

## Author contributions

FX, YL conceived and designed the experiments. CL, PW, MZ, LM performed the experiments. FX analyzed the data. FX wrote the paper.

### Conflict of interest statement

The authors declare that the research was conducted in the absence of any commercial or financial relationships that could be construed as a potential conflict of interest.

## References

[B1] Adams T. H. Boylan M. T. Timberlake W. E. (1988). *BrlA* is necessary and sufficient to direct conidiophore development in *Aspergillus nidulans*. Cell 54, 353–362. 10.1016/0092-8674(88)90198-5 3293800

[B2] Affeldt K. J. Brodhagen M. Keller N. P. (2012). *Aspergillus* oxylipin signaling and quorum sensing pathways depend on G protein-coupled receptors. Toxins 4, 695–717. 10.3390/toxins4090695 23105976 PMC3475224

[B3] Affeldt K. J. Carrig J. Amare M. G. Keller N. (2014). Global survey of canonical *Aspergillus flavus* G protein-coupled receptors. MBio 5, 1501–1514. 10.1128/mBio.01501-14 25316696 PMC4205791

[B4] Amaike S. Keller N. P. (2011). *Aspergillus flavus*. Annu. Rev. Phytopathol. 49, 107–133. 10.1146/annurev-phyto-072910-095221 21513456

[B5] Andrianopoulos A. Timberlake W. E. (1994). The *Aspergillus nidulans abaA* gene encodes a transcriptional activator that acts as a genetic switch to control development. Mol Cell Biol. 14, 2503–2515. 10.1128/MCB.14.4.2503 8139553 PMC358618

[B6] Bai Y. Wang S. Zhong H. Yang Q. Zhang F. Zhuang Z.. (2015). Integrative analyses reveal transcriptome-proteome correlation in biological pathways and secondary metabolism clusters in *A. flavus* in response to temperature. Sci. Rep. 5:14582. 10.1038/srep14582 26416011 PMC4586720

[B7] Baidya S. Duran R. M. Lohmar J. M. Harris-Coward P. Y. Cary J. W. Hong S.-Y.. (2014). VeA is associated with the response to oxidative stress in the aflatoxin producer *Aspergillus flavus*. Am. Soc. Microbiol. 13, 1095–1103. 10.1128/EC.00099-14 24951443 PMC4135802

[B8] Bennett J. W. Klich M. (2003). Mycotoxins. Clin. Microbiol. Rev. 16, 497–516. 10.1128/CMR.16.3.497-516.2003 12857779 PMC164220

[B9] Bluma R. V. Etcheverry M. G. (2008). Application of essential oils in maize grain: impact on *Aspergillus* section *Flavi* growth parameters and aflatoxin accumulation. Food Microbiol. 25, 324–334. 10.1016/j.fm.2007.10.004 18206775

[B10] Boylan M. T. Mirabito P. M. Willett C. E. Zimmerman C. R. Timberlake W. E. (1987). Isolation and physical characterization of three essential conidiation genes from *Aspergillus nidulans*. Mol. Cell Biol. 7, 3113–3118. 10.1128/MCB.7.9.3113 2823119 PMC367944

[B11] Caceres I. Khoury R. E. Bailly S. Oswald I. P. Puel O. Bailly J. D. (2017). Piperine inhibits aflatoxin B_1_ production in *Aspergillus flavus* by modulating fungal oxidative stress response. Fungal Genet. Biol. 107, 77–85. 10.1016/j.fgb.2017.08.005 28830793

[B12] Caceres I. Khoury R. E. Medina A. Lippi Y. Naylies C. Atoui A.. (2016). Deciphering the anti-aflatoxinogenic properties of eugenol using a large-scale q-PCR approach. Toxins 8:123. 10.3390/toxins8050123 27128940 PMC4885038

[B13] Calvo A. M. Bok J. Brooks W. Keller N. P. (2004). *VeA* is required for toxin and sclerotial production in *Aspergillus parasiticus*. Appl. Environ. Microbiol. 70, 4733–4739. 10.1128/AEM.70.8.4733-4739.2004 15294809 PMC492383

[B14] Chanda A. Roze L. V. Kang S. Artymovich K. A. Hicks G. R. Raikhel N. V.. (2009). A key role for vesicles in fungal secondary. Proc. Natl. Acad. Sci. U.S.A. 106, 19533–19538. 10.1073/pnas.0907416106 19889978 PMC2773199

[B17] Chang Y. C. Timberlake W. E. (1993). Identification of *Aspergillus brlA* response elements (BREs) by genetic selection in yeast. Genetics 133, 29–38. 8417986 10.1093/genetics/133.1.29 PMC1205295

[B15] Chang P. K. Abbas H. K. Weaver M. A. Ehrlich K. C. Scharfenstein L. L. Cotty P. J. (2012). Identification of genetic defects in the atoxigenic biocontrol strain *Aspergillus flavus* K49 reveals the presence of a competitive recombinant group in field populations. Int. J. Food Microbiol. 154, 192–196. 10.1016/j.ijfoodmicro.2012.01.005 22285533

[B16] Chang P.-K. Scharfenstein L. L. Luo M. Mahoney N. Molyneux R. J. Yu J.. (2010). Loss of *msnA*, a putative stress regulatory gene, in *Aspergillus parasiticus* and *Aspergillus flavus* increased production of conidia, aflatoxins and kojic acid. Toxins 3, 82–104. 10.3390/toxins3010082 22069691 PMC3210457

[B18] Chiaradia V. Paroul N. Cansian R. L. Júnior C. V. Detofol M. R. Lerin L. A.. (2012). Synthesis of eugenol esters by lipase-catalyzed reaction in solvent-free system. Appl. Biochem. Biotechnol. 168, 742–751. 10.1007/s12010-012-9814-5 22864649

[B19] Choi M.-J. Soottiantawat A. Nuchuchua O. Min S.-G. Ruktanonchai U. (2009). Physical and light oxidative properties of eugenol encapsulated by molecular inclusion and emulsion-diffusion method. Food Res. Int. 42, 148–156. 10.1016/j.foodres.2008.09.011

[B22] Cleveland T. E. Yu J. Fedorova N. Bhatnagar D. Payne G. A. Nieman W. C.. (2009). Potential of Aspergillus flavus genomics for applications in biotechnology. Trends Biotechnol. 27, 151–157. 10.1016/j.tibtech.2008.11.008 19195728

[B20] Cleveland T. E. Yu J. Bhatnagar D. Chen Z. Y. Brown R. L. Chang P. K. (2004). Progress in elucidating the molecular basis of the host plant *Aspergillus flavus* interaction, a basis for devising strategies to reduce aflatoxin contamination in crops. Toxin Rev. 23, 345–380. 10.1081/TXR-200027892

[B21] Clutterbuck A. J. (1969). A mutational analysis of conidial development in *Aspergillus nidulans*. Genetics 63, 317–327. 5366214 10.1093/genetics/63.2.317 PMC1212347

[B23] da Silva F. F. M. Monte F. J. Q. de Lemos T. L. G. do Nascimento P. G. G. de Medeiros Costa A. K. de Paiva L. M. M. (2018). Eugenol derivatives: synthesis, characterization, and evaluation of antibacterial and antioxidant activities. Chem. Cent. J. 12:34 10.1186/s13065-018-0407-4 PMC5880794 29611004

[B24] de Jong E. van Berkel W. J. H. van der Zwan R. P. de Bont J. A. M. (1992). Purification and characterization of vanillyl-alcohol oxidase from *Penicillinum simplicissimu*m: a novel aromatic alcohol oxidase containing covalently bound FAD. Eur. J. Biochem. 208, 651–657. 10.1111/j.1432-1033.1992.tb17231.x 1396672

[B25] Duran R. M. Cary J. W. Calvo A. M. (2007). Production of cyclopiazonic acid, aflatrem, and aflatoxin by *Aspergillus flavus* is regulated by *veA*, a gene necessary for sclerotial formation. Appl. Microbiol. Biotechnol. 73, 1158–1168. 10.1007/s00253-006-0581-5 16988822

[B26] FAO (1982). Evaluation of Certain Food Additives and Contaminants. Technical Report Series No. 20. FAO/WHO Expert Committee on Food Additives.

[B27] Furukawa H. Wieser M. Morita H. Sugio T. Nagasawa T. (1998). Purification and characterization of eugenol dehydrogenase from *Pseudomonas fluorescens* E118. Arch. Microbiol. 171, 37–43. 10.1007/s002030050675 9871017

[B28] Glisovic T. Bachorik J. L. Yong J. Dreyfuss G. (2008). RNA-binding proteins and post-transcriptional gene regulation. FEBS Lett. 582, 1977–1986. 10.1016/j.febslet.2008.03.004 18342629 PMC2858862

[B30] Grintzalis K. Vernardis S. I. Klapa M. I. Georgiou C. D. (2014). Role of oxidative stress in sclerotial differentiation and aflatoxin B_1_ biosynthesis in *Aspergillus flavus*. Appl. Environ. Microbiol. 80, 5561–5571. 10.1128/AEM.01282-14 25002424 PMC4178614

[B32] Groopman J. D. Kensler T. W. Wild C. P. (2008). Protective interventions to prevent aflatoxin-induced carcinogenesis in developing countries. Annu. Rev. Public Health 29, 187–203. 10.1146/annurev.publhealth.29.020907.090859 17914931

[B31] Han K.-H. Han K.-Y. Yu J.-H. Chae K.-S. Jahng K.-Y. Han D.-M. (2001). The nsdD gene encodes a putative GATA type transcription factor necessary for sexual development of Aspergillus nidulans. Mol. Microbiol. 41, 299–309. 10.1046/j.1365-2958.2001.02472.x 11489119

[B33] Han K.-H. Kin J.-H. Moon H. Kim S. Lee S. S. Han D. M.. (2008). The *Aspergillus nidulans esdC* (early sexual development) gene is necessary for sexual development and is controlled by *veA* and a heterotrimeric G protein. Fungal Genet. Biol. 45, 310–318. 10.1016/j.fgb.2007.09.008 17977758

[B35] Hanano A. Alkara M. Almousally I. Shaban M. Rahman F. Hassan M.. (2018). The peroxygenase activity of the *Aspergillus flavus* caleosin, AfPXG, modulates the biosynthesis of aflatoxins and their trafficking and extracellular secretion via lipid droplets. Front. Microbiol. 9:158. 10.3389/fmicb.2018.00158 29467750 PMC5808235

[B34] Hanano A. Almousally I. Shaban M. Blee E. (2015). A caleosin-like protein with peroxygenase activity mediates *Aspergillus flavus* development, aflatoxin accumulation, and seed infection. Appl. Envrion. Microbiol. 81, 6129–6144. 10.1128/AEM.00867-15 26116672 PMC4542254

[B36] Hoffmeister D. Keller N. P. (2007). Natural products of filamentous fungi: enzymes, genes, and their regulation. Nat. Prod. Rep. 24, 393–416. 10.1039/B603084J 17390002

[B37] Hong S. Y. Roze L. V. Wee J. Linz J. E. (2013). Evidence that a transcription factor regulatory network coordinates oxidative stress response and secondary metabolism in Aspergilli. Microbiologyopen 2, 144–160. 10.1002/mbo3.63 23281343 PMC3584220

[B38] Horchani H. Salem N. B. Zarai Z. Sayari A. Gargour Y. Chaâbouni M. (2010). Enzymatic synthesis of eugenol benzoate by immobilized *Staphylococcus aureus* lipase: optimization using response surface methodology and determination of antioxidant activity. Bioresour. Technol. 101, 2809–2817. 10.1016/j.biortech.2009.10.082 19969449

[B40] Hua S. S. Beck J. J. Sarreal S. B. L. Gee W. (2014). The major volatile compound 2-phenylethanol from the biocontrol yeast, *Pichia anomala*, inhibits growth and expression of aflatoxin biosynthetic genes of *Aspergillus flavus*. Mycotoxin Res. 30, 71–78. 10.1007/s12550-014-0189-z 24504634

[B39] Hua H. Xing F. Selvaraj J. N. Wang Y. Zhao Y. Zhou L.. (2014). Inhibitory effect of essential oils on *Aspergillus ochraceus* growth and ochratoxin A production. PLoS ONE 9:e108285. 10.1371/journal.pone.0108285 25255251 PMC4178002

[B41] International Agency for Research on Cancer (IARC) (1985). Summaries & Evaluations-Eugenol. 36, 75.

[B42] Isaac S. (1999). What is the mode of action of fungicides and how do fungi develop resistance? Mycologist 13, 38–39.

[B43] Jahanshiri Z. Shams-Ghahfarokhi M. Allameh A. Razzaghi-Abyaneh M. (2015). Inhibitory effect of eugenol on aflatoxin B_1_ production in *Aspergillus parasiticus* by downregulating the expression of major genes in the toxin biosynthetic pathway. World J. Microbiol. Biotechnol. 31, 1071–1078. 10.1007/s11274-015-1857-7 25896772

[B44] Jayashree T. Subramanyam C. (1999). Antiaflatoxigenic activity of eugenol is due to inhibition of lipid peroxidation. Lett. Appl. Microbiol. 28, 179–183. 10.1046/j.1365-2672.1999.00512.x 10196764

[B45] Jin J. Mazon H. van den Heuvel R. H. H. Janssen D. B. Fraaije M. W. (2007). Discovery of a eugenol oxidase from *Rhodococcus* sp. strain RHA1. FEBS J. 274, 2311–2321. 10.1111/j.1742-4658.2007.05767.x 17419730

[B46] Kanehisa M. Araki M. Goto S. Hattori M. Hirakawa M. Itoh M.. (2008). KEGG for linking genomes to life and the environment. Nucleic Acids Res. 36, D480–D484. 10.1093/nar/gkm882 18077471 PMC2238879

[B47] Karapinar M. (1990). Inhibitory effects of anethole and eugenol on the growth and toxin production of *Aspergillus parasiticus*. Int. J. Food Microbiol. 10, 193–199. 10.1016/0168-1605(90)90066-E 2397152

[B48] Kato N. Brooks W. Calvo A. M. (2003). The expression of sterigmatocystin and penicillin genes in *Aspergillus nidulans* is controlled by *veA*, a gene required for sexual development. Eukaryotic Cell 2, 1178–1186. 10.1128/EC.2.6.1178-1186.2003 14665453 PMC326641

[B49] Langmead B. Trapnell C. Pop M. Salzberg S. L. (2009). Ultrafast and memory-efficient alignment of short DNA sequences to the human genome. Genome Biol. 10:R25. 10.1186/gb-2009-10-3-r25 19261174 PMC2690996

[B50] Liang D. Xing F. Selvaraj J. N. Liu X. Wang L. Hua H.. (2015). Inhibitory effect of cinnamaldehyde, citral, and eugenol on aflatoxin biosynthetic gene expression and aflatoxin B_1_ biosynthesis in *Aspergillus flavus*. J. Food Sci. 80, M2917–M2924. 10.1111/1750-3841.13144 26556681

[B51] Lin J. Zhao X. Zhi Q. Zhao M. He Z. (2013). Transcriptomic profiling of *Aspergillus flavus* in response to 5-azacytidine. Fungal Genet. Biol. 56, 78–86. 10.1016/j.fgb.2013.04.007 23644151

[B52] Liu X. Guan X. Xing F. Lv C. Dai X. Liu Y. (2017). Effect of water activity and temperature on the growth of *Aspergillus flavus*, the expression of aflatoxin biosynthetic genes and aflatoxin production in shelled peanuts. Food Control 82, 325–332. 10.1016/j.foodcont.2017.07.012

[B53] Montibus M. Pinson-Gadais L. Richard-Forget F. Barreau C. Ponts N. (2013). Coupling of transcriptional response to oxidative stress and secondary metabolism regulation in filamentous fungi. Crit. Rev. Microbiol. 43, 1–14. 10.3109/1040841X.2013.829416 24041414

[B54] Mortazavi A. Williams B. A. McCue K. Schaeffer L. Wold B. (2008). Mapping and quantifying mammalian transcriptomes by RNA-Seq. Nat. Methods 5, 621–628. 10.1038/nmeth.1226 18516045 PMC13303166

[B55] Nam H. Kim M. M. (2013). Eugenol with antioxidant activity inhibits MMP-9 related to metastasis in human fibrosarcoma cells. Food Chem. Toxicol. 55, 106–112. 10.1016/j.fct.2012.12.050 23313798

[B29] OBrian G. R. Georgianna D. R. Wilkinson J. R. Yu J. Abbas H. K. Bhatnagar D. (2007). The effect of elevated temperature on gene transcription and aflatoxin biosynthesis. Mycologia 99, 232–239. 10.1080/15572536.2007.11832583 17682776

[B56] Opdyke D. L. J. (1975). Monographs on fragrance raw materials: eugenol. Food Cosm. Toxicol. 13, 545–547. 10.1016/0015-6264(75)90011-5 53171

[B57] Priebe S. Linde J. Albrecht D. Guthke R. Brakhage A. A. (2011). FungiFun: a webbased application for functional categorization of fungal genes and proteins. Fungal Genet. Biol. 48, 353–358. 10.1016/j.fgb.2010.11.001 21073976

[B58] Qi Y.-B. Wang X.-L. Shi T. Liu S. Xu Z.-H. Li X.. (2015). Multicomponent kinetic analysis and theoretical studies on the phenolic intermediates in the oxidation of eugenol and isoeugenol catalyzed by laccase. Phys. Chem. Chem. Phys. 17, 29597–29607. 10.1039/C5CP03475B 26477512

[B59] Reeve C. D. Carver M. A. Hopper D. J. (1989). The purification and characterization of 4-ethylphenol methylenehydroxylase, a flavocytochrome from *Pseudomonas putida* DJ1. Biochem. J. 263, 431–437. 10.1042/bj2630431 2556994 PMC1133447

[B60] Reverberi M. Fabbri A. A. Zjalic S. Ricelli A. Punelli F. Fanelli C. (2005). Antioxidant enzymes stimulation in *Aspergillus parasiticus* by *Lentinula edodes* inhibits aflatoxin production. Appl. Microbiol. Biotechnol. 69, 207–215. 10.1007/s00253-005-1979-1 15838675

[B61] Roze L. V. Koptina A. V. Laivenieks M. Beaudry R. M. Jones D. A. Kanarsky A. V.. (2011). Willow volatiles influence growth, development, and secondary metabolism in *Aspergillus parasiticus*. Appl. Microbiol. Biotechnol. 92, 359–370. 10.1007/s00253-011-3339-7 21614501

[B62] Satoh K. Sakagami H. Yokoe I. Kochi M. (1998). Interaction between eugenol-related compounds and radicals. Anticancer Res. 18, 425–428. 9568113

[B63] Sewall T. C. Mims C. W. Timberlake W. E. (1990). *AbaA* controls phialide differentiation in *Aspergillus nidulans*. Plant Cell 2, 731–739. 10.1105/tpc.2.8.731 2152124 PMC159926

[B64] Squire R. A. (1981). Ranking animal carcinogens: a proposed regulatory approach. Science 214, 877–880. 10.1126/science.7302565 7302565

[B65] Sun Q. Shang B. Wang L. Lu Z. Liu Y. (2015). Cinnamaldehyde inhibits fungal growth and aflatoxin B_1_ biosynthesis by modulating the oxidative stress response of *Aspergillus flavus*. Appl. Microbiol. Biotechnol. 100, 1355–1364. 10.1007/s00253-015-7159-z 26585445

[B66] Thomson E. Ferreira-Cerca S. Hurt E. (2013). Eukaryotic ribosome biogenesis at a glance. J. Cell Sci. 126, 4815–4821. 10.1242/jcs.111948 24172536

[B67] Timberlake W. E. Clutterbuck A. J. (1994). Genetic regulation of conidiation. Prog. Indust. Microbiol. 29, 383–427. 7765135

[B68] Trapnell C. Pachter L. Salzberg S. L. (2009). TopHat: discovering splice junctions with RNA-Seq. Bioinformatics 25, 1105–1111. 10.1093/bioinformatics/btp120 19289445 PMC2672628

[B69] Trapnell C. Williams B. A. Pertea G. Mortazavi A. Kwan G. van Baren M. J.. (2010). Transcript assembly and quantification by RNA-Seq reveals unannotated transcripts and isoform switching during cell differentiation. Nat. Biotechnol. 28, 511–515. 10.1038/nbt.1621 20436464 PMC3146043

[B70] Tsitsigiannis D. I. Keller N. P. (2006). Oxylipins act as determinants of natural product biosynthesis and seed colonization in *Aspergillus nidulans*. Mol. Microbiol. 59, 882–892. 10.1111/j.1365-2958.2005.05000.x 16420358

[B71] Tsitsigiannis D. I. Keller N. P. (2007). Oxylipins as developmental and host-fungal communication signals. Trends Microbiol. 15, 109–118. 10.1016/j.tim.2007.01.005 17276068

[B72] Velluti A. Sanchis V. Ramos A. Egido J. Marin S. (2003). Inhibitory effect of cinnamon, clove, lemongrass, oregano and palmarose essential oils on growth and fumonisin B_1_ production by *Fusarium proliferatum* in maize grain. Int. J. Food Microbiol. 89, 145–154. 10.1016/S0168-1605(03)00116-8 14623380

[B73] Wang Z. Gerstein M. Snyder M. (2009). RNA-Seq: a revolutionary tool for transcriptomics. Nat. Rev. Genet. 10, 57–63. 10.1038/nrg2484 19015660 PMC2949280

[B74] Wei D. Zhou L. Selvaraj J. N. Zhang C. Xing F. Zhao Y.. (2014). Molecular characterization of atoxigenic *Aspergillus flavus* isolates collected in China. J. Microbiol. 52, 559–565. 10.1007/s12275-014-3629-8 24879349

[B75] Wilhelm B. T. Marguerat S. Watt S. Schubert F. Wood V. Goodhead I.. (2008). Dynamic repertoire of a eukaryotic transcriptome surveyed at single-nucleotide resolution. Nature 453, 1239–1243. 10.1038/nature07002 18488015

[B76] Wright M. S. Greene-McDowelle D. M. Zeringue H. J. Bhatnagar D. Cleveland T. E. (2000). Effects of volatile aldehydes from *Aspergillus*-resistant varieties of corn on *Aspergillus parasiticus* growth and aflatoxin biosynthesis. Toxicon 38, 1215–1223. 10.1016/S0041-0101(99)00221-4 10736475

[B77] Wu F. (2014). Perspective: time to face the fungal threat. Nature 516, S7–S7. 10.1038/516S7a 25470199

[B78] Xing F. Wang L. Liu X. Selvaraj J. N. Wang Y. Zhao Y.. (2017). Aflatoxin B_1_ inhibition in *Aspergillus flavus* by *Aspergillus niger* through down regulating expression of major biosynthetic genes and AFB_1_ degradation atoxigenic *A. falvus*. Int. J. Food Microbiol. 256, 1–10. 10.1016/j.ijfoodmicro.2017.05.013 28578264

[B79] Yu J. Chang P. K. Ehrlich K. C. Cary J. W. Bhatnagar D. Cleveland T. E.. (2004). Clustered pathway genes in aflatoxin biosynthesis. Appl. Environ. Microbiol. 70, 1253–1262. 10.1128/AEM.70.3.1253-1262.2004 15006741 PMC368384

[B80] Yu J. Fedorova N. D. Montalbano B. G. Bhatnagar D. Cleveland T. E. Bennett J. W.. (2011). Tightcontrol of mycotoxin biosynthesis gene expression in *Aspergillus flavus* by temperature as revealed by RNA-Seq. FEMS Microbiol. Lett. 322, 145–149. 10.1111/j.1574-6968.2011.02345.x 21707733

[B81] Yu J.-H. Keller N. (2005). Regulation of secondary metabolism in filamentous fungi. Annu. Rev. Phytopathol. 43, 437–458. 10.1146/annurev.phyto.43.040204.140214 16078891

[B82] Zhang F. Guo Z. Zhong H. Wang S. Yang W. Liu Y.. (2014). RNA-Seq-Based transcriptome analysis of aflatoxigenic *Aspergillus flavus* in response to water activity. Toxins 6, 3187–3207. 10.3390/toxins6113187 25421810 PMC4247253

