# Supplementary material for: Large-Scale Comparative Analysis of Eugenol-Induced/Repressed Genes Expression in Aspergillus flavus Using RNA-seq
Source: Front Microbiol. 2018 May 30;9:1116. doi: 10.3389/fmicb.2018.01116 (PMC5988903; doi:10.3389/fmicb.2018.01116)
Supplement: Supplementary file 1 [file Table_1.DOCX]

Table S1. Characteristics of strain *A. flavus* YC-15

| Item | Characteristics |
| --- | --- |
| Origin | Peanut-cropped soil |
| Collection site | Yicheng, Hubei Province, P. R. China |
| Year | 2011 |
| Conidia | Green conidia after 3.5 days on the modified rose bengal agar (M-RB) |
| Aflatoxin production | AFB_1_ |
| Toxigenic ability | Middle |
| Sclerotia characterization | Large sclerotia, >400μm in diameter |
| Genbank accession number for calmodulin gene | AY974340.1 |
| Species name | *Aspergillus flavus* |

Table S2. Transcriptional activity of some possible eugenol-converting enzyme genes

| Gene ID  (AFLA_x) | Untreated (FPKM) | D125 (FPKM) | Log | Annotated_gene_function |
| --- | --- | --- | --- | --- |
| 004360 | 0.11 | 0.26 | 1.21 | Alcohol dehydrogenase |
| 005070 | 0.09 | 0.15 | 0.70 | Alcohol dehydrogenase |
| 008880 | 0.21 | 0.35 | 0.72 | Zinc-containing alcohol dehydrogenase |
| 010050 | 0.24 | 0.38 | 0.69 | Alcohol dehydrogenase |
| 024270 | 0.66 | 0.86 | 0.38 | Aryl-alcohol dehydrogenase |
| 024700 | 0.24 | 1.07 | 2.15 | Short-chain alcohol dehydrogenase |
| 038770 | 1.04 | 1.31 | 0.33 | Alcohol dehydrogenase |
| 128700 | 0.22 | 0.32 | 0.54 | Alcohol dehydrogenase |
| 000890 | 0.91 | 1.24 | 0.44 | Laccase |
| 123160 | 4.74 | 9.31 | 0.97 |  |
| 016150 | 1.48 | 4.72 | 1.67 | Lipase |
| 020170 | 0.44 | 1.17 | 1.40 | Extracellular lipase |
| 057690 | 0.15 | 0.63 | 2.08 | Lipase/acylhydrolas |
| 058010 | 0.80 | 1.48 | 0.89 | Lipase/acylhydrolase |
